# Regulation of dopamine release by CASK-*β* modulates locomotor initiation in *Drosophila melanogaster*

**DOI:** 10.3389/fnbeh.2014.00394

**Published:** 2014-11-18

**Authors:** Justin B. Slawson, Elena A. Kuklin, Konark Mukherjee, Nicolás Pírez, Nathan C. Donelson, Leslie C. Griffith

**Affiliations:** Department of Biology, Volen Center for Complex Systems, National Center for Behavioral Genomics, Brandeis UniversityWaltham, MA, USA

**Keywords:** *Drosophila melanogaster*, neurotransmitter release, CASK, dopamine, locomotion, Hsc70-4

## Abstract

CASK is an evolutionarily conserved scaffolding protein that has roles in many cell types. In *Drosophila*, loss of the entire *CASK* gene or just the *CASK-β* transcript causes a complex set of adult locomotor defects. In this study, we show that the motor initiation component of this phenotype is due to loss of CASK-*β* in dopaminergic neurons and can be specifically rescued by expression of CASK-*β* within this subset of neurons. Functional imaging demonstrates that mutation of *CASK-β* disrupts coupling of neuronal activity to vesicle fusion. Consistent with this, locomotor initiation can be rescued by artificially driving activity in dopaminergic neurons. The molecular mechanism underlying this role of CASK-*β* in dopaminergic neurons involves interaction with Hsc70-4, a molecular chaperone previously shown to regulate calcium-dependent vesicle fusion. These data suggest that there is a novel CASK-*β*-dependent regulatory complex in dopaminergic neurons that serves to link activity and neurotransmitter release.

## Introduction

Dopamine is a biogenic amine present in most nervous systems. Its neuromodulatory function is highly conserved across species, contributing to complex biological states and behaviors, including locomotion, attention, learning, sleep, and mood (Costa, 2007; Palmiter, 2008). Not surprisingly, misregulation of dopaminergic signaling can lead to the development of a number of different human pathologies, the most common of which is Parkinson’s Disease, a neurodegenerative condition characterized by the progressive loss of dopaminergic cells within the substantia nigra (Braak and Del Tredici, 2008). Loss of dopaminergic cells has been directly linked to aberrations in motor function, including tremor, rigidity, bradykinesia, and postural instability (Lees et al., 2009). Attempts to characterize the disease at the cellular level have suggested that excitotoxicity, mitochondrial dysfunction, oxidative stress, and protein misfolding/aggregation are all important pathophysiological features (Schapira, 2009; Vaarmann et al., 2013), but the primary cause of the disease has remained elusive (Obeso et al., 2010). The vast majority of cases are idiopathic and may stem from heightened sensitivity of the dopaminergic system to inflammation and environmental toxins/stressors, possibly in conjunction with unidentified genetic factors (Thomas and Beal, 2007; Surmeier et al., 2010).

One interesting feature of the pathology observed in Parkinson’s disease is that despite the fact that the entire brain is subjected to the same genetic and environmental factors, only the dopaminergic neurons appear to be especially vulnerable. For this reason, it has been suggested that there are likely to be cell-specific risk factors that predispose dopaminergic cells to dysfunction (Obeso et al., 2010; Surmeier et al., 2010). Despite this, however, little work has been done to elucidate differences between dopaminergic cells and other neurons that are relevant to this phenomenon.

The function of dopamine in locomotion is highly conserved across species, and insects also show a Parkinsonian-like motor initiation deficit upon disruption of this neurotransmitter system (Feany and Bender, 2000; Riemensperger et al., 2011). In this study, we demonstrate that CASK-*β*, a *Drosophila* scaffolding protein orthologous to mammalian CASK, regulates motor initiation by facilitating transmitter release specifically in dopaminergic cells. Previous studies have implicated CASK-*β* in proper locomotor behavior (Martin and Ollo, 1996; Sun et al., 2009; Slawson et al., 2011). The severity of the motor phenotype resulting from loss of CASK-*β* is striking, and appears to influence several different locomotor parameters, including motor initiation, motor maintenance, speed, and acceleration. Recent work (Slawson et al., 2011) mapped the spatial requirement for CASK-*β* to a discrete subset of cells within the fly central nervous system that did not include major known premotor neuron groups.

Using directed expression via the GAL4/UAS system (Fischer et al., 1988; Brand and Perrimon, 1993; Phelps and Brand, 1998), we show here that a number of cells within this subset are dopaminergic neurons, and that reconstitution of CASK-*β* specifically in these cells selectively rescues the motor initiation component of the mutant’s complex locomotor phenotype. The motor initiation disruption in *CASK-β* mutants likely stems from aberrations in vesicle release at dopaminergic synapses, which may be driven by a recently identified interaction between CASK-*β* and the molecular chaperone Hsc70-4 (Mukherjee et al., 2014). Interestingly, while this interaction appears to be present in multiple cell types throughout the invertebrate nervous system, our results indicate that the association between these two proteins is only behaviorally relevant to locomotion in dopaminergic cells. Hsc70-4 and its homologs have been implicated in mammalian models of Parkinson’s disease (Auluck et al., 2002; Pemberton et al., 2011) and their levels are altered in brain samples from human patients (Alvarez-Erviti et al., 2010; Sala et al., 2014). Whether regulation of dopamine release by an Hsc4/CASK interaction is relevant to mammalian dopaminergic function is unknown, but our results suggest that this may be an interesting new avenue of inquiry.

## Materials and methods

### Fly stocks and maintenance

All flies were raised at 25°C in a 12 h:12 h light:dark cycle unless otherwise stated. Flies were fed standard cornmeal-dextrose agar media. *UAS-CASK-β-YFP* and its domain deletions were subcloned into EcoRI/XhoI sites of a PhiC31 vector. DNA was sent to Rainbow Transgenic Flies, Inc. (Camarillo, CA, USA) for embryonic injections into fly strain #24872 (Genotype: *M{vas-int.Dm}ZH-2A, PBac{y[+]-attP-3B}8VK00037*) to produce flies with targeted insertions on the second chromosome. Transformants were selected by eye color based on the presence of the mini-white gene. All other fly lines were obtained from the Bloomington Stock Center (Bloomington, IN, USA), except for *TH-Gal4* (from Jay Hirsh, University of Virginia, Charlottesville, USA), *C164-Gal4* (from Vivian Budnik, University of Massachusetts, Worcester, MA, USA), *dilp2-Gal4* (from Ulrike Heberlein, Janelia Farms), *TH-Gal80* (from Julie Simpson, Janelia Farms), *UAS-dTRPA1* (from Paul Garrity, Brandeis University, Waltham, USA), *UAS-synaptopHluorin* (from Gero Miesenböck, University of Oxford, UK), *Hsc70-4* Δ ^19^ (a strong hypomorphic allele from Konrad Zinsmaier, University of Arizona, Tucson, AZ, USA), *tub-GAL80*^ts^ (Ron Davis, Scripps Florida) and the full-length *UAS-CASK-β* line, which was *UAS-CASK 10.20* (from Peter Bryant, University of California, Irvine, CA, USA). For behavioral rescue experiments, all GAL4 and UAS- lines were crossed into the *CASK-β*^P18^ null background (Slawson et al., 2011) to ensure that endogenous protein wouldn’t confound the tissue-specific reconstitution of CASK-*β*.

### P-element mobilization

The previously described *TH-Gal4* line (Friggi-Grelin et al., 2003) demonstrated locomotor abnormalities without the addition of any GAL4-responsive transgenes (data not shown). As this could make it difficult to properly quantify changes in movement following the reconstitution of CASK, we utilized *P*-element excision to mobilize the *TH-Gal4* construct to a new chromosomal location, which we have named *THump-Gal4*. To generate *THump-Gal4*, the third chromosome *TH-Gal4* *P-element* construct was mobilized using standard genetic techniques (Greenspan, 1997). Briefly, *TH-Gal4* flies were crossed with w;+/CyOΔ2-3;+/TM6B flies. F1 male progeny were mated with Df(1)w females, and the resulting F2 progeny were selected for *P*-element mobilization based on retention of both eye color and the humoral trait associated with TM6B. The resulting insertion was mapped to the second chromosome, and expresses GAL4 in the same sets of dopaminergic cells observed in the original *TH-Gal4* line (see Supplemental Figure S1).

### Confocal imaging

Adult brains were dissected in phosphate buffered saline (PBS), fixed for 15 min in 4% paraformaldehyde, washed in PBT (0.5% Triton X-100 in PBS), stained in primary antibodies at 4°C for *ca*. 48 h, washed in PBT, stained in fluorescent secondary antibodies at 4°C for overnight, then washed in PBT and mounted using Vectashield (Vector Laboratories, Inc.). A Leica TCS SP5 confocal microscope was used to capture sequential images of the brains at 20× magnification with LAS AF imaging software from Leica (version 2.6.0). Anti-TH mouse monoclonal antibody used in these experiments was obtained from Immunostar (Hudson, WI, USA), and was used at a concentration of 1:500. Anti-GFP rabbit polyclonal antibody was procured from Santa Cruz Biotechnology, Inc., and was also used at a concentration of 1:500. Alexa633 anti-mouse antibody and alexa488 anti-rabbit antibody (Invitrogen) were both used as secondary antibodies at 1:200. All antibodies were diluted in PBT+10% normal goat serum (NGS Jackson ImmunoResearch Laboratories).

### Locomotor analysis

High-resolution video tracking was performed as described (Slawson et al., 2009, 2011). Briefly, mated male flies aged 1–3 days were sorted into groups of 10 under light CO_2_ anesthesia. Following at least a 48 h recovery at 25°C, flies at 4–6 days of age were gently knocked into a translucent observation chamber. Following a 30 min acclimation period, the trial was initiated by the administration of a brief pulse of air, and fly movement was video-recorded for 30 s. Ten seconds prior to the administration of the air pulse, the chambers were given five gentle taps on a padded surface to wake the flies up for testing. Locomotor traces of individual flies were recorded using DIAS 3.2 software (Soll, 1995; Soll et al., 2001) and analyzed using custom-designed scripts in Matlab (Mathworks, Natick, MA, USA). In the behavioral rescue experiments, all measurements for UAS- or Gal4- containing lines were normalized to control flies by dividing each individual average by the mean of wild type flies for a given parameter. In these cases, wild type performance for each parameter is denoted by a dotted black line in the bar graphs. At least seven trials over three or more days were performed for each genotype. For experiments using dTRPA1, flies were grown and maintained at 23°C. Three hours before behavioral testing, flies were shifted to 27°C and subsequently assayed at this temperature. For experiments utilizing Gal80^ts^, flies were grown and maintained at 18°C until sorting for behavioral analysis. During the 48 h recovery window preceding behavioral testing, flies were shifted to 25°C and assayed at this temperature. In all cases, locomotor chambers were pre-equilibrated to the testing temperature for at least 30 min.

### SynaptopHluorin imaging

Imaging experiments were performed using a naked brain preparation. Briefly, fly brains are dissected out of the head of the animal and placed in a perfusion chamber (Harvard Apparatus) immediately before the experiments. Brains were allowed to recover in the perfusion chamber before recording started. The preparation was under constant perfusion with artificial hemolymph-like (AHL) saline flowing by gravity feed at approximately 3–4 ml/min. The AHL contains 5 mM HEPES, 4 mM NaHCO_3_ pH 7.5, 108 mM NaCl, 5 mM KCl, 2 mM CaCl_2_, 8.2 mM MgCl_2_, 1 mM NaH_2_PO_4_, 5 mM Trehalose, 10 mM sucrose (Wang et al., 2003). We recorded each brain for a period of 240 s (2 Hz frequency). Brains were stimulated by perfusion of 5 mM carbachol (carbamoylcholine chloride, Sigma) for 30 s by switching between reservoirs with a manually controlled 3-way solenoid. All experiments were performed using an Olympus BX51WI microscope and a 60× (0.9 NA) water immersion lens (Olympus LUMPlanFl), using the following filter set: Exciter HQ470/40×, Dichroic Q495LP, Emitter HQ525/50 m. The acquisition was performed using the open-source software µManager (Edelstein et al., 2010), and recordings are saved using a CCD camera (Hammamatsu Orca C472—80-12AG). Data were analyzed offline using custom software written in ImageJ (NIH), Matlab (Mathworks) and Excel (Microsoft).

### Immunoblots

Heads from frozen male and female flies were removed by vortexing in liquid nitrogen and homogenized in 1× SDS buffer. Protein samples were separated by SDS-PAGE and transferred to nitrocellulose. Immunoblots were visualized using either ECL or Luminol detection reagents (GE Healthcare) on film (developed with a Kodak X-OMAT 200A Developer). The polyclonal antibody used to visualize CASK (Marble et al., 2005) was a kind gift from Gisela Wilson (University of Wisconsin, Madison, WI, USA). Green fluorescent protein and actin monoclonal antibodies were obtained commercially (Roche and Millipore, respectively). All primary antibodies were used at a concentration of 1:1000. Horseradish peroxidase-conjugated anti-guinea pig (Jackson Laboratories) and anti-mouse (GE healthcare) secondary antibodies were used at a concentration of 1:5000.

### Statistics

All statistical analysis was done using either JMP software (SAS Institute, Cary, NC, USA) or Microsoft Excel. For all imaging experiments, significance was determined using a two-tailed Student’s *t*-test (*P* < 0.05). For all behavioral manipulations, one-way analysis of variance (ANOVA) was computed, and a Tukey HSD (honest significance difference) test was used for *post hoc* analysis as previously reported (Slawson et al., 2011). Error bars in all figures represent the standard error of the mean (SEM), with significant differences between groups indicated by different letters (conditions that are not statistically different from one another are labeled with the same letter) or asterisks (*α* < 0.05).

## Results

### Expression of CASK-*β* in dopaminergic neurons rescues motor initiation defects

**Figure 1:**
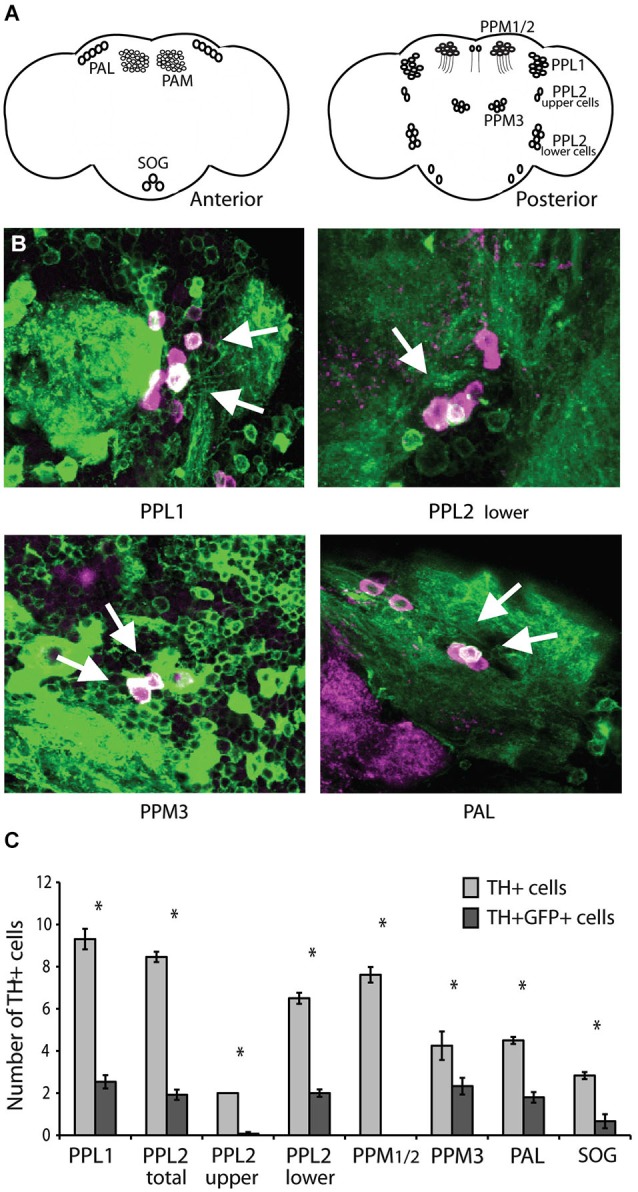
***C164-Gal4* expresses in dopaminergic neurons. (A)** Dopaminergic neurons in the *Drosophila* brain are organized into distinct clusters (Mao and Davis, 2009). Left panel shows anterior brain clusters. Right panel shows clusters in the posterior brain. **(B)** The *C164-Gal4* expression pattern visualized by crossing the GAL4 line to *UAS-mCD8GFP*. Brains were co-stained with anti-TH (shown in purple) to reveal dopaminergic neurons, while visualization of GFP expression was enhanced with an anti-GFP antibody (shown in green). The two staining patterns are presented as an overlay, and the arrows indicate points of overlap between the two expression patterns. **(C)** The number of TH+ and TH+GFP+ double positive cells in each dopaminergic neuron cluster were counted. The *C164-Gal4* driver expresses in specific cells within the PAL, PPL1, PPL2, and PPM3 clusters. Significant differences between TH+ and TH+GFP+ double positive staining are represented by asterisks (*P* < 0.05, Student’s *t*-test). See Section Methods for details on statistical tests.

Expression of CASK-*β* under control of the *C164-Gal4* driver line can rescue motor behavior in *CASK-β* mutants (Slawson et al., 2011). This driver line expresses in a discrete subset of CNS neurons, many of which are difficult to identify based on morphology and position alone. None of the known motor control centers, including central complex regions (Strauss and Heisenberg, 1993; Martin et al., 1999; Strauss, 2002) and mushroom bodies (Martin et al., 1998) are strongly represented in the *C164-Gal4* pattern (Slawson et al., 2011). Although it is interesting that these brain regions are not a direct site of action for CASK-*β* in locomotion, this does not rule out the possibility that CASK-*β* affects the function of one of these known motor centers indirectly. One way that this could occur is if CASK acted in modulatory dopaminergic neurons, which have been implicated in a number of motor disorders (reviewed in Piccini, 2004), and also directly innervate the central complex (Kong et al., 2010; White et al., 2010) and mushroom bodies (Neckameyer, 1998; Mao and Davis, 2009; White et al., 2010). Indeed, dissecting the expression pattern of *C164-Gal4* revealed the presence of dopaminergic neurons from several different cell clusters (Figure 1). Intriguingly, a number of the dopaminergic cells identified map to the PPL1 cluster (Figures 1B,C), which is an important site of degeneration in many fly models of Parkinson’s Disease which share similar locomotor abnormalities (Whitworth et al., 2005; Trinh et al., 2008).

**Figure 2:**
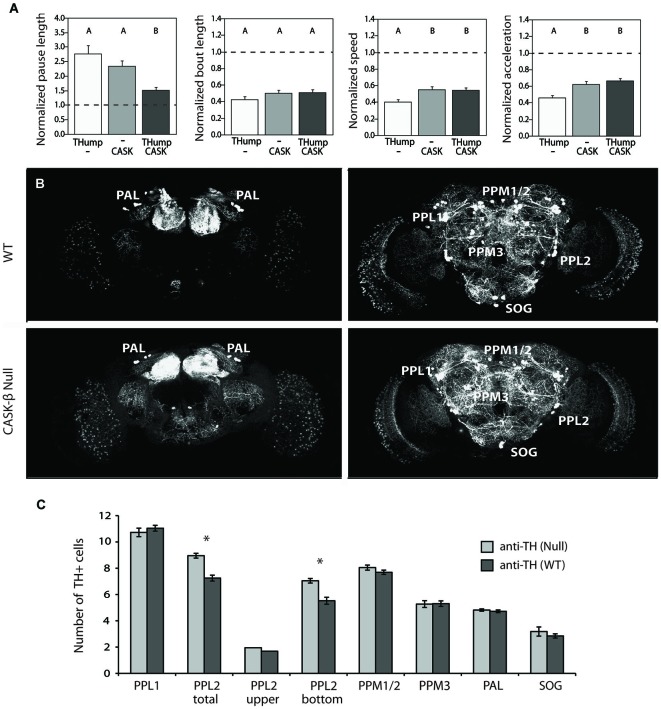
**CASK-*β* contributes to dopaminergic control of motor initiation without disrupting the neuronal architecture of dopaminergic cell clusters**. **(A)** Ectopic expression of CASK-*β* in dopaminergic neurons using *Thump-Gal4* significantly rescues the pause length parameter of locomotor behavior, which is a measure of motor initiation. The other metrics of locomotion remained unchanged. Data from all groups are normalized to WT control levels that are shown as a dotted black line at 1.0 on the Y-axis of each graph. The magnitude of the deviation from the dotted line is proportional to severity of phenotype. Transgenes present in each group of animals are indicated below the histogram bars: “THump” = *THump-Gal4*, and “CASK” = *UAS-CASK-β*. All transgenes were on a *CASK-β* mutant background. Letters indicate significant differences between groups (*P* < 0.05, ANOVA with Tukey HSD). See Section Methods for details on statistical tests. **(B)** Dopaminergic neurons were visualized with an anti-TH antibody using confocal imaging. **(C)** Cell counts of both WT brains (top panels, anterior and posterior are shown) and CASK-*β* null brains (bottom panels, anterior and posterior are shown) demonstrate equivalent numbers of dopaminergic neurons across most dopaminergic cell clusters between these two genotypes. Significant differences between WT and *CASK-β* mutant are represented by asterisks (*P* < 0.05, Student’s *t*-test).

To directly assess the hypothesis that dopaminergic cells are involved in the *CASK-β* locomotor phenotype, we attempted to rescue the behavior in a null animal by reconstituting CASK-*β* specifically in these neurons using the GAL4/UAS binary expression system (Fischer et al., 1988; Brand and Perrimon, 1993; Phelps and Brand, 1998). Targeted expression within these cells was achieved through use of a new GAL4 line called *THump-Gal4*, which drives expression specifically in tyrosine hydroxylase-positive cells (Supplemental Figure S1). Interestingly, expression of CASK-*β* with *THump-Gal4* significantly rescued motor behavior, but only in the “Pause Length” parameter, which is a measure of motor initiation. The other three behavioral metrics previously shown to be affected in the null animals (Slawson et al., 2011) remained unchanged (Figure 2A). In addition, ectopic expression of CASK-*β* with the *C164-Gal4* driver line failed to rescue the pause length parameter when dopaminergic expression was simultaneously repressed using *TH-Gal80* (Supplemental Figure S2). These results indicate that individual components of the locomotor phenotype are the result of CASK-*β* deficiency in different neuroanatomical loci within the C164-GAL4^+^ neurons of the central nervous system, suggesting that these are independently controlled behavioral parameters.

Abnormalities in dopaminergic signaling have been shown to produce similar motor initiation deficits in the fly (Riemensperger et al., 2011), and degeneration of dopamine-producing cells is believed to underlie locomotor abnormalities in *Drosophila* models of Parkinson’s Disease (reviewed in Muñoz-Soriano and Paricio, 2011). For this reason, we assessed the dopaminergic system of wild type and CASK-*β* mutants to see if there were any significant differences in cell numbers. Interestingly, none of the dopaminergic cell groups showed significant reductions in the mutant (Figures 2B,C). In contrast, loss of CASK-*β* led to a small, but significant increase in TH^+^ cells within the PPL2 cluster (Figure 2C). This implies that the motor initiation phenotype does not stem from loss of dopaminergic cells. The cause for the small increase in TH^+^ cells in the mutant is unknown, but could be due to differences in genetic background or a role for CASK-*β* in repression of TH expression in a small population of neurons.

### CASK-*β* mutants demonstrate a functional deficit in dopamine release

**Figure 3:**
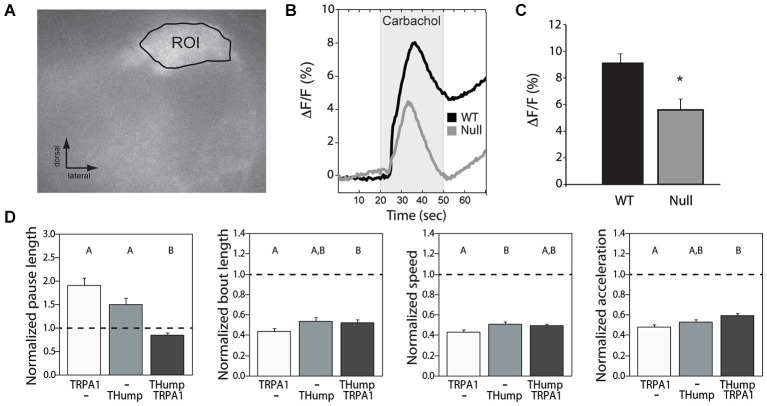
**Synaptic vesicle release deficits underlie the dopaminergic motor initiation phenotype. (A)** SynaptopHluorin was used to visualize vesicle release from dopaminergic cells of the antennal lobes using a naked brain preparation. An example region of interest is shown. **(B)** Upon stimulation with carbachol, *CASK-β* mutants displayed a reduced synaptopHluorin response indicative of less vesicle fusion. **(C)** Average peak response was significantly different between WT and *CASK-β* mutants (*P < 0.05, Student’s *t*-test). **(D)** Increasing synaptic activity in dopaminergic neurons of *CASK-β* mutant flies via dTRPA1 activation specifically rescued the pause length parameter of locomotion, but failed to cause a change in the other three metrics of motor behavior. Data from all groups are normalized to WT control levels which are shown as a dotted black line at 1.0 on the Y-axis of each graph. The magnitude of the deviation from the dotted line is proportional to severity of phenotype. Transgenes present in each group of animals are indicated below the histogram bars: “THump” = *THump-Gal4*, and “TRPA1” = UAS-dTRPA1. As above, all transgenes were on a *CASK-β* mutant background. Letters indicate significant differences between groups (*P* < 0.05, ANOVA with Tukey HSD). See Section Methods for details on statistical tests.

Previous work at the larval neuromuscular junction has suggested that *Drosophila* CASK affects locomotor behavior through modulation of synaptic vesicle release (Zordan et al., 2005; Chen and Featherstone, 2011), most probably by altering calcium responsiveness (Gillespie and Hodge, 2013). To address the issue of whether or not synaptic vesicle release underlies the adult dopaminergic locomotor phenotype, we performed optical imaging experiments using flies that express the pH-sensitive sensor synaptopHluorin (SpH) under control of *THump-Gal4*. This sensor reports synaptic vesicle fusion to the membrane by means of a membrane bound pH-sensitive variant of GFP (Miesenböck et al., 1998; Ng et al., 2002). SynaptopHluorin is quenched in the acidic environment of the vesicular lumen, while highly fluorescent in the higher pH encountered within the synaptic cleft (Miesenböck et al., 1998). Changes in fluorescence thus act as a measure of vesicle release following synaptic activity or stimulation. For these experiments, we expressed SpH in dopaminergic cells of either a wild type or *CASK-β* null animal and measured changes in fluorescence within a glomerulus of the antennal lobe (a region with dense dopaminergic innervation) using a naked brain preparation (Figure 3A). Since acetylcholine is the major excitatory neurotransmitter in the insect brain (Leech and Sattelle, 1993), we stimulated neurons by applying 5 mM carbachol, a cholinergic agonist. We found that the peak response elicited by carbachol was significantly decreased in the dopaminergic terminals of null flies compared with wild type brains (Figures 3B,C). This finding suggests that loss of CASK-*β* results in a functional deficit of synaptic output at dopaminergic synapses.

To determine whether simply increasing the activity level of dopaminergic neurons was sufficient to rescue the locomotor deficit seen in these flies, we hyperactivated dopaminergic neurons using dTRPA1, a warmth-gated non-specific cation channel (Hamada et al., 2008). Activation of these channels in dopaminergic neurons of *CASK-β* null flies by increasing temperature significantly rescued the “Pause Length” parameter, but had little effect on the other three indices (Figure 3D). This suggests that the motor initiation deficit observed in the null flies stems largely from a reduction in synaptic output from dopaminergic cells. These data also indicate that the reduction in SpH signal amplitude likely results from a quantitative difference in vesicle release, as opposed to a lack of dopamine production, pointing to a problem in coupling of neuronal activity to vesicle fusion.

### Initiation of motor behavior is dependent on the isoform-specific N-terminus of CASK-*β*

Mammalian CASK appears to play a general role in vesicle release through a neurexin-dependent mechanism (Hata et al., 1996; Atasoy et al., 2007) and this has been posited to be important in *Drosophila* as well (Sun et al., 2009). In order to interact with neurexin, the N-terminal CaMK-like and L27 domains of mammalian CASK first bind with Mint1/X11 and Veli, respectively (Butz et al., 1998). This interaction is believed to modulate synaptic vesicle release in two ways. First, the N-terminal CaMK-like domain of CASK displays pseudokinase activity under certain conditions, and has been shown to phosphorylate neurexin *in vivo* (Mukherjee et al., 2008). Secondly, the tripartite complex of CASK, Mint1/X11, and Veli is capable of recruiting additional proteins to the presynaptic terminal, which indirectly influences neurexin-mediated exocytosis (Butz et al., 1998; Missler and Sudhof, 1998). For both of these mechanisms, the N-terminus of CASK is required for proper modulation of vesicle release from axon terminals.

**Figure 4:**
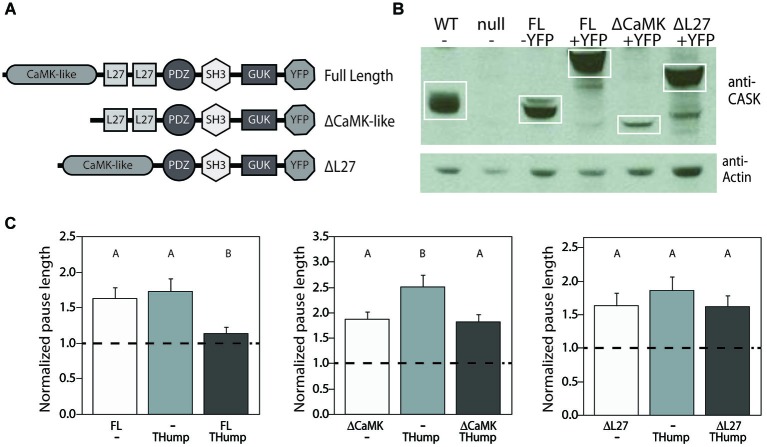
**N-terminal domains of CASK-*β* are required for behavioral rescue in dopaminergic cells. (A)** Domain structures of CASK-*β* proteins used for rescue. In-frame deletions were generated in the UAS-CASK-*β*-YFP construct to ablate either the CaMK-like or L27 domains. **(B)** CASK-*β*-YFP proteins were expressed using a pan-neuronal driver line (*C155-Gal4*) and visualized via immunoblot of fly heads. Actin was immunoblotted as a loading control. **(C)** Expression of full length CASK-*β*-YFP with *THump-Gal4* rescued pause length, while both domain-truncated CASK-*β* proteins failed to rescue the locomotor deficit when expressed under control of this driver. Data from all groups are normalized to WT control levels which are shown as a dotted black line at 1.0 on the Y-axis of each graph. The magnitude of the deviation from the dotted line is proportional to severity of phenotype. Transgenes present in each group of animals are indicated below the histogram bars: “FL” = Full Length CASK-*β*, “ΔCaMK” = CaMK-like domain-ablated CASK-*β*, and “ΔL27” = L27 domain-ablated CASK-*β*. All transgenes were on a *CASK-β* mutant background, and letters indicate significant differences between groups (*P* < 0.05, ANOVA with Tukey HSD). See Section Methods for details on statistical tests.

Based on the high degree of homology between the N-terminal regions of mammalian CASK and *Drosophila* CASK-*β*, we set out to determine whether or not the CaMK-like and L27 domains are required for motor initiation in the fly. We generated three new YFP-tagged CASK-*β* transgenes, each under the control of GAL4-responsive upstream activating sequence (UAS): two truncated forms (one missing the CaMK-like domain, the other without the L27 domains), and a full-length version (depicted in Figure 4A and visualized via immunoblot in Figure 4B). As expected, when expressed in dopaminergic cells, the full-length CASK-*β*-YFP significantly rescued motor initiation behavior as compared with mutant controls (Figure 4C). Both the CaMK-like and L27 deleted forms of CASK-*β*-YFP, however, failed to significantly rescue initiation (Figure 4C). While all three of these constructs express CASK-*β*, the ΔCaMK-like domain ablated line appears to have reduced expression, suggesting that this domain could also contribute to the overall stability of the protein (Figure 4B), but makes interpretation of the failure to rescue less clear. The expression level of the ΔL27 CASK protein, however, was similar to wild type, indicating that the L27 domains of the isoform-specific N-terminus are necessary for behavioral rescue. This is supportive of the idea that something analogous to the mammalian tripartite complex could be central to the role of CASK-*β* in regulating output of dopaminergic neurons. Alternatively, it is possible that some other protein, also requiring interactions with the N-terminal domain, is acting as a partner for CASK in regulation of dopaminergic neuron vesicle fusion. There are many candidates since CASK, as a member of the MAGUK family of scaffolding proteins, has been shown to have a multitude of binding partners (Zheng et al., 2011).

### CASK-*β* interacts genetically with Hsc70-4, a regulator of synaptic vesicle release, to regulate locomotion

Because CASK has so many potential binding partners, it is important to have a methodology to identify putative cell-specific interactors. Recently, we developed a method for mass spectrometry identification of co-immunoprecipitated proteins bound to full length CASK-*β*-YFP expressed in defined neuronal populations using the GAL4/UAS system (Mukherjee et al., 2014). This published data set allowed us to obtain a list of cell-specific binding partners for CASK-*β*. We found that while CASK may participate in a physical complex with neurexin in some neuronal cell-types, this interaction was not observed in dopaminergic neurons (Mukherjee et al., 2014), suggesting the possibility that there might be other mechanisms by which CASK can regulate vesicle fusion.

One candidate that was present in CASK-*β* pull downs from dopaminergic neurons was Hsc70-4 (also known as HSC4). At the larval neuromuscular junction, Hsc70-4 has been shown to be required for the coupling of activity to neurotransmitter release (Bronk et al., 2001). The exocytosis phenotype of the *Hsc70-4* mutant at this synapse can be rescued by enhancing calcium influx, which is similar to our finding that driving activity in dopaminergic neurons is able to rescue the *CASK-β* locomotor initiation defect.

**Figure 5:**
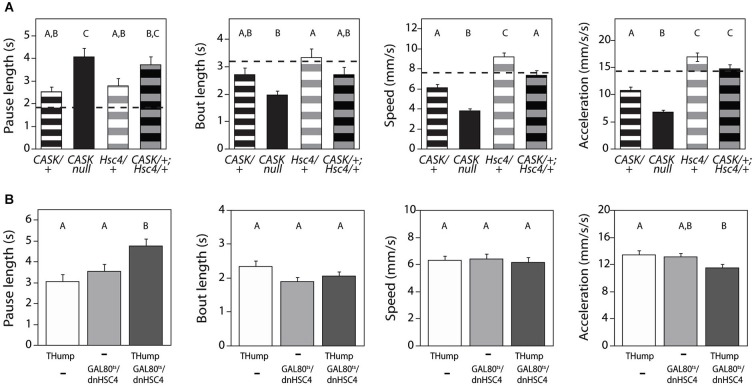
***CASK-β* interacts behaviorally with** ***Hsc70-4***. **(A)** Flies heterozygous for a hypomorphic *Hsc70-4* mutation *(Hsc4/+)* show a defect in the locomotor pause length parameter similar to that of *CASK-β*/+ flies, but less severe than CASK-*β* homozygous null flies. Pause length is significantly worse in transheterozygote flies containing one mutant allele of each gene *(CASK-β/+;Hsc4/+)*, indicating that the two genes likely interact in regulating motor initiation. *Hsc70-4*/+ flies do not on their own present a phenotype in the other three measured parameters of locomotion, nor do they appear to worsen these parameters when combined with the *CASK-β* mutation. Here the behavioral performance of WT flies is presented as a dotted black line, with the magnitude of the deviation from the dotted line proportional to severity of phenotype. Genotypes of each group of animals are indicated below the histogram bars: “*CASK*” = *CASK-β^P18^*, a null allele; “*Hsc4*” = *Hsc70-4 Δ^19^*, a hypomorphic allele; “*+*” = a WT allele. **(B)** A dominant negative Hsc70-4 transgene was expressed under control of *THump-Gal4* and expression limited to adulthood using *tub-Gal80*^ts^. Here “Thump” = *THump-Gal4* and “Gal80^ts^/dnHSC4” = *tub-Gal80^ts^/UAS-[dn]Hsc70-4*. Expression of this dominant negative construct in adult dopaminergic cells results in reduced motor initiation behavior (as evidenced by the change in the pause length parameter), but no significant changes in the other indices of locomotor behavior. As before, letters indicate significant differences between groups (*P* < 0.05, ANOVA with Tukey HSD). See Section Methods for details on statistical tests.

To test the idea that CASK and Hsc70-4 might cooperate to regulate locomotor initiation, we examined the genetic interactions between *CASK-β* and *Hsc70-4*. We first looked at behavior in transheterozygotes of *CASK-β^P18^* (a null allele) and *Hsc70-4* Δ^19^ (a strong hypomorph). As previously reported, *CASK-β* null mutants show gene dose-dependent deficits in multiple locomotor parameters. Interestingly, *Hsc70-4* heterozygotes are only defective in “Pause Length”, a metric for motor initiation (Figure 5A) but were normal for other parameters, suggesting that Hsc70-4 has a specific role in motor initiation. Homozygotes of this genotype did not survive to adulthood and therefore could not be assayed. Transheterozygotes, animals missing one copy of the *CASK-β* gene and one copy of *Hsc70-4*, showed a synthetic phenotype worse than either heterozygote alone for motor initiation. In the case of the other parameters, the transheterozygote behaved more similarly to the *CASK-β* heterozygote (Figure 5A), suggesting no synthetic effect and little contribution to phenotype from loss of a single copy of *Hsc70-4*.

These data were consistent with an interaction of CASK-*β* and Hsc70-4 regulating dopamine release, but since the effects of germline mutations are not limited to dopaminergic neurons, the motor initiation phenotype of the *Hsc70-4* mutant could be the result of actions of this gene during development and/or in non-dopaminergic neurons. To test the temporal and spatial specificity of the interaction, we utilized a temperature-sensitive GAL80 (McGuire et al., 2004) to limit *THump-Gal4*-driven dominant negative Hsc70-4 (Elefant and Palter, 1999) to adult neurons. Expression of dominant negative Hsc70-4 only in adult dopaminergic cells produced a significant motor initiation phenotype but did not affect other locomotor parameters (Figure 5B). These data are supportive of an acute role for Hsc70-4 in regulation of dopaminergic neuron function.

## Discussion

### CASK-*β* regulates initiation of movement by affecting the function of dopaminergic neurons

The ability to initiate locomotion is critical to an organism’s ability to perform other more complex behavioral tasks necessary for survival. As such, the mechanisms involved in generating and maintaining proper motor initiation are regulated at many different biological levels. CASK has clearly been shown to have a role in governing these processes (Martin and Ollo, 1996; Sun et al., 2009; Slawson et al., 2011). In this study we demonstrate that one aspect of locomotor behavior, initiation of movement, is dependent on the presence of CASK-*β* in dopaminergic neurons. Interestingly, other facets of the *CASK-β* null locomotor phenotype were not rescued by expression of CASK-*β* in this subset of neurons, but required much broader expression of CASK-*β* (Slawson et al., 2011). The finding that there is independent control of functional locomotor parameters is quite novel. Future studies involving cell-specific rescue of these parameters could lead to identification of new motor circuit elements. Since CASK-*β* has been shown to interact with unique repertoires of proteins across different cell types (Mukherjee et al., 2014), our results suggest the possibility that the complex and multi-faceted behavioral phenotype of the null mutant is due to distinct molecular complexes being disrupted in these different groups of cells.

### A novel role for CASK-*β* in regulation of neurotransmitter release in adult dopaminergic neurons

While loss of the entire *CASK* gene can disrupt evoked release at both larval and adult neuromuscular junctions (Zordan et al., 2005; Sun et al., 2009; Chen and Featherstone, 2011), there has been less work on the role of CASK at adult central synapses. A study on the role of *CASK-β* in learning (Malik et al., 2013) demonstrated a decrease in calcium responsiveness of the mutant in mushroom bodies. Here we show that in *CASK-β* mutants, vesicle fusion induced by a cholinergic agonist is reduced in dopaminergic neurons. These data suggest a model in which the defect in motor initiation is due to abnormal coupling of neuronal activity with neurotransmitter release. Consistent with this hypothesis, driving activity at very high levels in dopaminergic neurons with dTRPA1 appears to rescue initiation without affecting other locomotor parameters in *CASK-β* mutants. This argues for a function role of CASK-*β* in dopamine release.

Neurexin was a likely candidate partner for CASK-*β* as a regulator of neurotransmitter release (Hata et al., 1996; Mukherjee et al., 2008; Sun et al., 2009). Previous work had profiled cell-specific CASK-*β* protein complexes by identifying proteins present in immunoprecipitations of tagged CASK-*β* expressed in particular cell types on a null mutant genetic background (Mukherjee et al., 2014). In that study, neurexin was identified as a binding partner in pull-downs when tagged CASK-*β* was driven in all neurons, but when the pull-downs were repeated from dopaminergic neurons, neurexin was not present. There were, however, a number of other proteins known to be involved in neurotransmitter release which were enriched in dopaminergic neurons relative to other populations, including Comatose (the *Drosophila* NSF homolog), Synapsin, and Hsc70-4. The presence of Synapsin was reduced in pull-downs using tagged versions of CASK-*β* lacking either the CaMK or L27 domain, suggesting that this might be a critical interaction, since CASK-*β* lacking these domains fails to rescue motor initiation.

The most potentially interesting candidate from the list of interacting proteins, however, was Hsc70-4. At the *Drosophila* third instar larval neuromuscular junction, this protein has been shown to modulate the coupling of neuronal activity and calcium to neurotransmitter release (Bronk et al., 2001). Defects in neurotransmitter release in hypomorphic *Hsc70-4* mutants can be rescued by increasing extracellular calcium or by high frequency stimulation, which transiently increases resting calcium in the presynaptic terminal. This was very reminiscent of our results (Figure 3) demonstrating that driving activity in dopaminergic neurons with dTRPA1 can rescue the locomotor initiation phenotype of *CASK-β* mutants. This similarity suggested that the interaction with Hsc70-4 might be functionally important for the *CASK-β* null phenotype. Genetic interactions between *CASK-β* and both *Hsc70-4* mutants and a dominant negative Hsc70-4 transgene strongly supported this hypothesis.

There was a surprising amount of functional specificity for dopaminergic neurons in this interaction given that the protein association between CASK and Hsc70-4 was seen in multiple neuron types (Mukherjee et al., 2014). *Hsc70-4/+* heterozygous mutants had defects only in the initiation parameter of locomotion, but not in the other locomotor metrics (Figure 5). These other parameters are controlled by independent circuits that involve non-dopaminergic neurons. While it seems likely that the calcium responses in these circuits are also blunted in the *CASK-β* mutant (Malik et al., 2013), their dysfunction does not seem to involve an Hsc70-4-sensitive pathway since reduction of gene copy number has no effect on them and did not enhance their *CASK-β*/+ phenotype. This could indicate that they have other non-Hsc70-4-dependent mechanisms for coupling release to calcium or that the disruption of the function of these circuits in the *CASK-β* mutant does not involve changes in neurotransmitter release.

The molecular details of how Hsc70-4 and CASK-*β* regulate the vesicle fusion machinery remain to be elucidated. There are many possibilities, several of which involve changes in localization of release-regulating proteins to enhance their access to calcium. The mechanism is likely to be complex, but our data indicate it may have special relevance in dopaminergic neurons.

### Other roles for CASK-*β* in dopaminergic neurons?

Since CASK has many binding partners, it is possible that this protein does more in dopaminergic neurons than just regulate neurotransmitter release. It has been recently reported that this protein also associates with mitochondria (Mukherjee et al., 2014), which are a known site of dysfunction in Parkinson’s Disease. Analysis of CASK-*β* binding partners (Mukherjee et al., 2014) also identified Dj1-*β*, a recessive cause of familial Parkinson’s disease in humans, as a dopaminergic neuron-specific binding partner. Investigation of the role of CASK in age-related aspects of dopaminergic neuron function may provide some insights into the specific cell biology of these neurons.

## Conflict of interest statement

The authors declare that the research was conducted in the absence of any commercial or financial relationships that could be construed as a potential conflict of interest.

## Supplementary material

Figure S1 ***TH-Gal4***** and** ***THump-Gal4***** express in all dopaminergic neurons in all adult brain dopaminergic clusters**. **(A)** The expression pattern of *TH-Gal4* was assessed by crossing it to *UAS-mCD8GFP*. Brains from these animals were dissected, fixed, stained with anti-TH and anti-GFP, and visualized using confocal imaging. Cell counts demonstrate that all TH+ cells in the PPL1, PPL2, PPM1/2, PPM3, PAL, and SOG clusters were present in the *TH-Gal4* expression pattern, since virtually all TH+ cells were also GFP+. **(B)** The same approach was used to map dopaminergic cells within the *THump-Gal4* expression pattern. Like *TH-Gal4*, *THump-Gal4* also expresses in all TH+ cells.

Figure S2 **Loss of dopaminergic cells from the *C164-Gal4* expression pattern blocks rescue**. Behavioral rescue experiments using *C164-Gal4* to drive ectopic expression of CASK-*β* in the central nervous system was repeated as in Slawson et al. (2011). In this instance, activity of the GAL4 driver in dopaminergic cells was repressed by the presence of *TH-Gal80*. Preventing expression of CASK-*β* in these cells prevented the behavioral rescue of pause length, further implicating these cells as the cellular locus for CASK-*β* in motor behavior. “CASK” = *UAS-CASK-β*, “THGal80” = *TH-Gal80*, and “C164” = *C164-Gal4*. Data from all groups are normalized to WT control levels, which are shown as a dotted black line at 1.0 on the Y-axis of each graph. The magnitude of the deviation from the dotted line is proportional to severity of phenotype. All transgenes were on a *CASK-β* mutant background, and letters indicate significant differences between groups (*P* < 0.05, ANOVA with Tukey HSD). See Methods for details on statistical tests.
