# Supplementary figures and images for: Regulation of dopamine release by CASK-β modulates locomotor initiation in Drosophila melanogaster
Source: Front Behav Neurosci. 2014 Nov 18;8:394. doi: 10.3389/fnbeh.2014.00394 (PMC4235261; doi:10.3389/fnbeh.2014.00394)

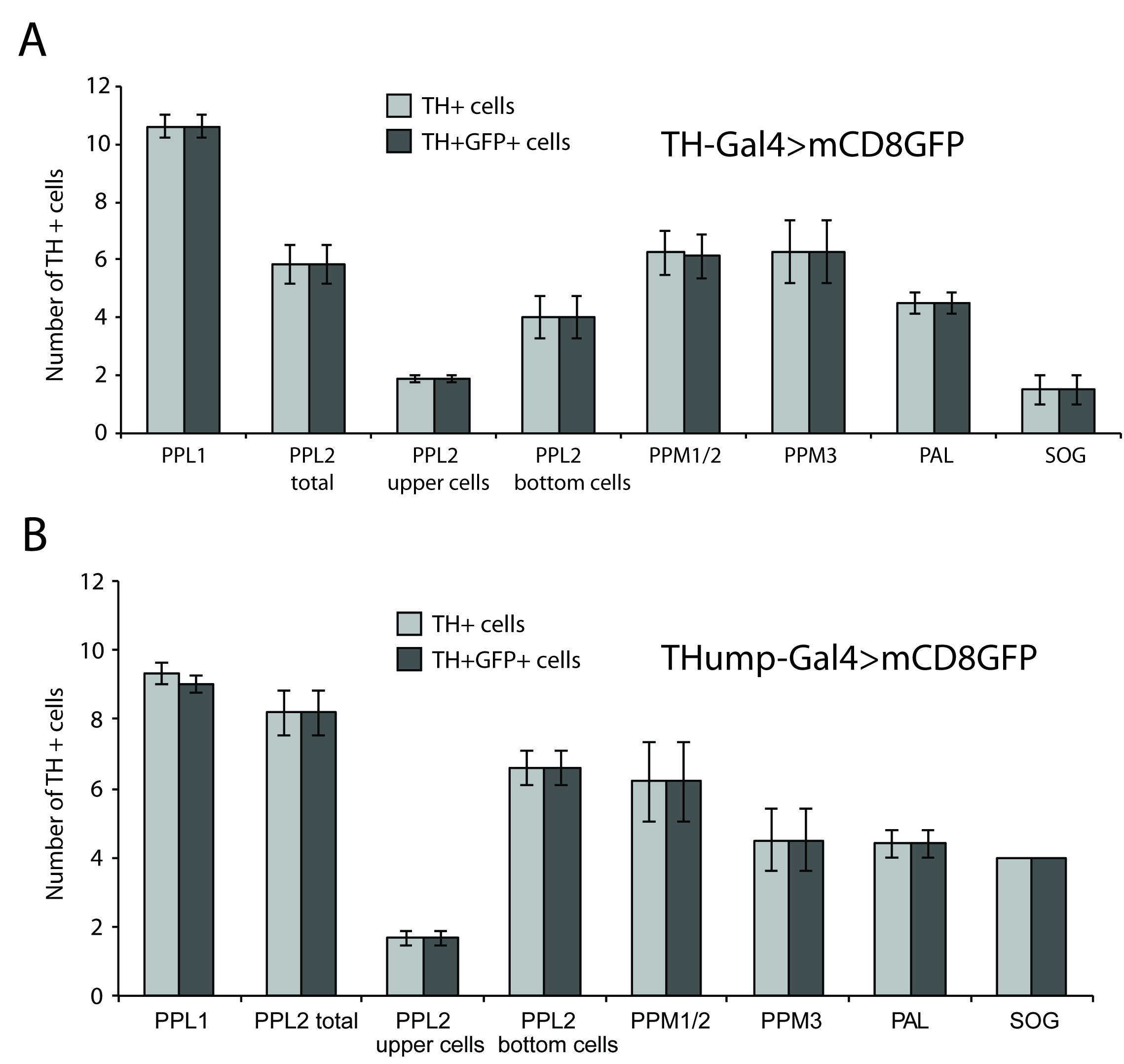

Supplement: Figure S1 — TH-Gal4 and THump-Gal4 express in all dopaminergic neurons in all adult brain dopaminergic clusters. (A) The expression pattern of TH-Gal4 was assessed by crossing it to UAS-mCD8GFP. Brains from these animals were dissected, fixed, stained with anti-TH and anti-GFP, and visualized using confocal imaging. Cell counts demonstrate that all TH+ cells in the PPL1, PPL2, PPM1/2, PPM3, PAL, and SOG clusters were present in the TH-Gal4 expression pattern, since virtually all TH+ cells were also GFP+. (B) The same approach was used to map dopaminergic cells within the THump-Gal4 expression pattern. Like TH-Gal4, THump-Gal4 also expresses in all TH+ cells. [file Image_1.JPEG]

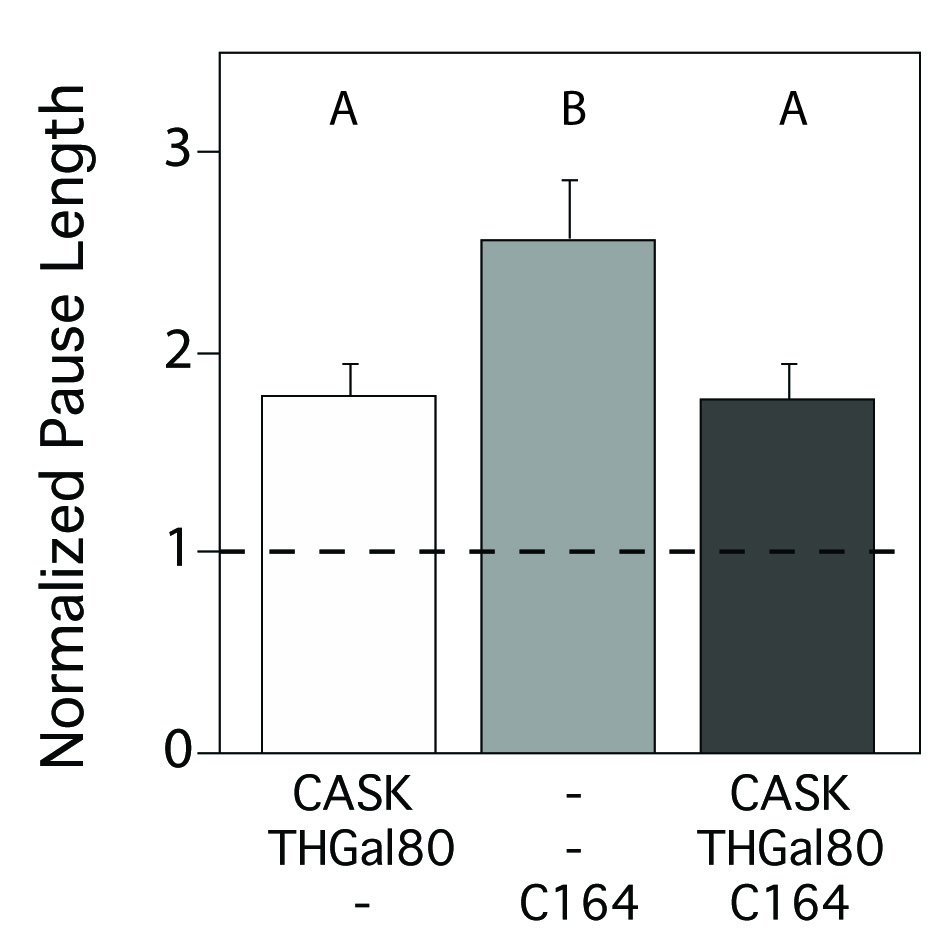

Supplement: Figure S2 — Loss of dopaminergic cells from the C164-Gal4 expression pattern blocks rescue. Behavioral rescue experiments using C164-Gal4 to drive ectopic expression of CASK-β in the central nervous system was repeated as in Slawson et al. (2011). In this instance, activity of the GAL4 driver in dopaminergic cells was repressed by the presence of TH-Gal80. Preventing expression of CASK-β in these cells prevented the behavioral rescue of pause length, further implicating these cells as the cellular locus for CASK-β in motor behavior. “CASK” = UAS-CASK-β, “THGal80” = TH-Gal80, and “C164” = C164-Gal4. Data from all groups are normalized to WT control levels, which are shown as a dotted black line at 1.0 on the Y-axis of each graph. The magnitude of the deviation from the dotted line is proportional to severity of phenotype. All transgenes were on a CASK-β mutant background, and letters indicate significant differences between groups (P < 0.05, ANOVA with Tukey HSD). See Methods for details on statistical tests. [file Image_2.JPEG]
